# 含全身放射治疗预处理方案异基因造血干细胞移植治疗成人急性淋巴细胞白血病的疗效分析

**DOI:** 10.3760/cma.j.cn121090-20230822-00084

**Published:** 2024-03

**Authors:** 倩倩 肖, 晓林 于, 晓晨 宋, 怡西 侯, 磊 邓, 文君 李, 芳 周

**Affiliations:** 联勤保障部队第九六〇医院血液病科，济南 250031 Department of Hematology, The 960^th^ Hospital of The People's Liberation Army (PLA) Joint Logistics Support Force, Jinan 250031, China

**Keywords:** 全身放射治疗, 急性淋巴细胞白血病, 造血干细胞移植, 预处理方案, Total body irradiation, Acute lymphocytic leukemia, Stem cell transplantation, Conditioning regimen

## Abstract

**目的:**

比较含全身放射治疗（TBI）和仅基于化疗预处理方案的异基因造血干细胞移植（allo-HSCT）治疗成人急性淋巴细胞白血病（ALL）的疗效，探讨影响预后的相关因素。

**方法:**

收集2015年1月至2022年8月于联勤保障部队第九六〇医院血液病科接受allo-HSCT治疗的95例成人ALL患者的临床资料，根据预处理方案不同分为TBI/环磷酰胺组（TBI/Cy组，53例）和白消安/环磷酰胺组（Bu/Cy组，42例）。比较两组在造血重建、移植相关并发症、移植后复发率、非复发死亡率（NRM）、总生存（OS）率、无白血病生存（LFS）率等方面的差异，并对预后影响因素进行分析。

**结果:**

TBI/Cy组、Bu/Cy组中性粒细胞植入中位时间分别为14（10～25）d、14（10～24）d（*P*＝0.106），血小板植入中位时间分别为17（10～42）d、19（11～42）d（*P*＝0.488）；Ⅱ～Ⅳ度急性移植物抗宿主病（aGVHD）累积发生率分别为41.5％、35.7％（*P*＝0.565），Ⅲ/Ⅳ度aGVHD累积发生率分别为24.5％、4.8％（*P*＝0.009），重度慢性移植物抗宿主病（cGVHD）的发生率分别为16.7％、13.5％（*P*＝0.689）；巨细胞病毒（CMV）血症发生率分别为41.5％、35.7％（*P*＝0.565），EB病毒血症发生率分别为34.0％、35.7％（*P*＝0.859），重度感染发生率分别为43.4％、33.3％（*P*＝0.318），出血性膀胱炎发生率分别为20.8％、50.0％（*P*＝0.003）。TBI/Cy组、Bu/Cy组中位随访时间分别为37.1、53.3个月，移植后2年累积复发率分别为17.0％、42.9％（*P*＝0.017），NRM分别为24.5％、7.1％（*P*＝0.120），LFS率分别为58.5％、50.0％（*P*＝0.466），OS率分别为69.8％、64.3％（*P*＝0.697）。多因素分析显示，预处理方案含TBI是allo-HSCT后复发的保护性因素（*HR*＝0.304，95％ *CI* 0.135～0.688，*P*＝0.004），而对NRM的影响不显著（*HR*＝1.393，95％ *CI* 0.355～5.462，*P*＝0.634）；感染是成人ALL患者allo-HSCT后OS的独立危险因素。

**结论:**

含TBI预处理方案与基于化疗预处理方案allo-HSCT治疗成人ALL比较，Ⅲ/Ⅳ度aGVHD发生率较高，出血性膀胱炎发生率及复发率较低。

## Abstract (en)

**Objective:**

To analyze the efficacy of allo-HSCT with total body irradiation（TBI）and chemotherapy alone in the treatment of adult ALL and to explore the factors affecting prognosis.

**Methods:**

The clinical data of 95 adult patients with ALL who underwent allo-HSCT from January 2015 to August 2022 were included. According to the conditioning regimen, the patients were divided into two groups: the TBI plus cyclophosphamide（TBI/Cy）group（*n*＝53）and the busulfan plus cyclophosphamide（Bu/Cy）group（*n*＝42）. Hematopoietic reconstitution after transplantation, GVHD, transplantation-related complications, relapse rate（RR）, non-relapse mortality（NRM）, OS, and LFS were compared, and the factors related to prognosis were analyzed.

**Results:**

The median time of neutrophil engraftment was 14（10−25）days in the TBI/Cy group and 14（10−24）days in the Bu/Cy group（*P*＝0.106）. The median time of megakaryocyte engraftment was 17（10−42）days in the TBI/Cy group and 19（11–42）days in the Bu/Cy group（*P*＝0.488）. The incidence of grade Ⅱ−Ⅳ acute GVHD（aGVHD）in the TBI/Cy and Bu/Cy groups was 41.5% and 35.7%, respectively（*P*＝0.565）. The incidence of grade Ⅲ−Ⅳ aGVHD in these two groups was 24.5% and 4.8%, respectively（*P*＝0.009）. The incidence of severe chronic GVHD in the two groups was 16.7% and 13.5%, respectively（*P*＝0.689）. The incidence of cytomegalovirus infection, Epstein-Barr virus infection, severe infection, and hemorrhagic cystitis in the two groups was 41.5% and 35.7%（*P*＝0.565）, 34.0% and 35.7%（*P*＝0.859）, 43.4% and 33.3%（*P*＝0.318）, and 20.8% and 50.0%（*P*＝0.003）, respectively. The median follow-up time was 37.1 months and 53.3 months in the TBI/Cy and Bu/Cy groups, respectively. The 2-year cumulative RR was 17.0% in the TBI/Cy group and 42.9% in the Bu/Cy group（*P*＝0.017）. The 2-year cumulative NRM was 24.5% and 7.1%, respectively（*P*＝0.120）. The 2-year LFS was 58.5% and 50.0%, respectively（*P*＝0.466）. The 2-year OS rate was 69.8% and 64.3%, respectively（*P*＝0.697）. In the multivariate analysis, the conditioning regimen containing TBI was a protective factor for relapse after transplantation（*HR*＝0.304, 95% *CI* 0.135−0.688, *P*＝0.004）, whereas the effect on NRM was not significant（*HR*＝1.393, 95% *CI* 0.355−5.462, *P*＝0.634）. Infection was an independent risk factor for OS after allo-HSCT in adult patients with ALL.

**Conclusion:**

allo-HSCT based on TBI conditioning regimen had lower relapse rate and lower incidence of hemorrhagic cystitis for adult ALL, compared with chemotherapy regimen. While the incidence o grade Ⅲ/Ⅳ aGVHD was hgher in TBI conditioning regimen than that in chemotherapy regimen.

急性淋巴细胞白血病（ALL）是成人最为常见的急性白血病类型之一，占成人急性白血病的20％～40％[1]。成人ALL患者通常化疗后复发率很高、预后极差，allo-HSCT仍是唯一治愈手段[2]–[3]。预处理是allo-HSCT的重要组成部分，是影响移植疗效和预后的关键。全身放射治疗（TBI）联合环磷酰胺（TBI/Cy）和白消安联合环磷酰胺（Bu/Cy）是两种广泛使用的ALL移植前清髓性预处理（MAC）方案。研究表明，含TBI预处理方案较化疗预处理方案具有显著优势，能降低疾病复发率，提高总生存（OS）率和无病生存（DFS）率[4]–[6]。但也有研究显示两种方案移植后OS和DFS相似[7]–[10]。本回顾性研究旨在比较TBI/Cy和Bu/Cy两种预处理方案allo-HSCT治疗成人ALL的疗效和安全性。

## 病例与方法

一、一般资料

本研究纳入2015年1月至2022年8月于联勤保障部队第九六〇医院血液病科行allo-HSCT治疗的95例成人ALL患者。根据预处理方案分为TBI/Cy组（53例）和Bu/Cy组（42例）。TBI/Cy组中位年龄27（18～53）岁，Bu/Cy组中位年龄25（18～58）岁。所有患者诊断分型采用WHO 2016标准[11]。

二、供者选择及移植方式

供者选择顺序一般为同胞全相合供者、≥9/10相合的无关供者、单倍体供者。根据相关专家共识[12]，优先考虑年轻男性供者。同胞全相合造血干细胞移植（MSD-HSCT）、单倍体造血干细胞移植（haplo-HSCT）及无关供者造血干细胞移植（UD-HSCT）分别为24、69、2例；47例患者行骨髓移植（BMT）联合外周血干细胞（PBSCT），48例行PBSCT。

三、干细胞动员及采集

移植前4 d开始给予粒细胞集落刺激因子5～10 µg·kg^−1^·d^−1^皮下注射，动员供者造血干细胞，移植前1 d采集供者骨髓，移植当天采集供者外周血干细胞。若首次采集的细胞数未达标准，则次日追加采集一次。

四、预处理方案

1. MSD-HSCT及UD-HSCT：①TBI/Cy：TBI总剂量9～10 Gy（分3 d），环磷酰胺60 mg·kg^−1^·d^−1^静脉滴注，×2 d；②Bu/Cy：白消安3.2 mg·kg^−1^·d^−1^静脉滴注，×4 d，环磷酰胺60 mg·kg^−1^·d^−1^静脉滴注，×2 d。

2. haplo-HSCT：①TBI/Cy+Ara-C：TBI总剂量9～10 Gy（分3 d），环磷酰胺1.8 g·m^−2^·d^−1^静脉滴注，×2 d；阿糖胞苷2 g/m^2^静脉滴注，每12 h 1次，×2 d；②Bu/Cy+Ara-C：白消安3.2 mg·kg^−1^·d^−1^静脉滴注，×3 d；环磷酰胺1.8 g·m^−2^·d^−1^静脉滴注，×2 d；阿糖胞苷2 g/m^2^静脉滴注，每12 h 1次，×2 d。

五、移植物抗宿主病（GVHD）预防

MSD-HSCT患者采用环孢素A+短程甲氨蝶呤+霉酚酸酯方案，haplo-HSCT及UD-HSCT患者采用环孢素A+短程甲氨蝶呤+霉酚酸酯+兔抗人胸腺细胞免疫球蛋白（rATG）方案。

六、疗效评价及随访

连续3 d外周血中性粒细胞绝对值≥0.5×10^9^/L为粒细胞植入；连续7 d血小板计数≥20×10^9^/L且脱离血小板输注为血小板植入。aGVHD的诊断和分级采用Glucksberg西雅图标准[13]，cGVHD诊断及分级参照美国国立卫生研究院（NIH）标准[14]–[15]。OS为从PBSC输注之日起到任何原因导致死亡的时间，DFS为从PBSC输注之日起到出现疾病复发的时间（以首次出现的事件为准）或任何原因导致死亡的时间。复发为移植后骨髓形态学检查发现骨髓中原始细胞超过5％或外周血出现原始细胞或发生髓外浸润。

七、统计学处理

应用SPSS 25.0统计学软件进行统计分析，计量资料使用Mann-Whitney *U*检验；计数资料使用卡方检验或使用Fisher精确概率法。长生存、复发及NRM均通过Kaplan-Meier法估算，率的比较使用Log-rank检验或竞争风险模型。应用Cox回归模型进行单因素和多因素分析，单因素分析中*P*<0.1的指标纳入多因素分析。*P*<0.05认为差异有统计学意义。

## 结果

一、一般资料

TBI/Cy组和Bu/Cy组患者在基本临床特征上差异均无统计学意义（均*P*>0.05），详见表1。

**表1:** 两种异基因造血干细胞移植预处理方案急性淋巴细胞白血病患者的基本临床特征比较

基本特征	Bu/Cy组（42例）	TBI/Cy组（53例）	统计量	*P*值
性别［例（%）］			*χ^2^*＝2.962	0.085
男	28（66.7）	26（49.1）		
女	14（33.3）	27（50.9）		
患者年龄［例（%）］			*χ^2^*＝0.032	0.859
≥35岁	15（35.7）	18（34.0）		
<35岁	27（64.3）	35（66.0）		
疾病分型［例（%）］			*χ^2^*＝2.673	0.102
T-ALL	6（14.3）	15（28.3）		
B-ALL	36（85.7）	38（71.7）		
费城染色体［例（%）］			*χ^2^*＝0.244	0.621
阳性	10（23.8）	15（28.3）		
阴性	32（76.2）	38（71.7）		
诊断时高白细胞血症［例（%）］			*χ^2^*＝0.209	0.647
是	21（50.0）	24（45.3）		
否	21（50.0）	29（54.7）		
髓外浸润病史［例（%）］			*χ^2^*＝2.426	0.119
有	11（26.2）	22（41.5）		
无	31（73.8）	31（58.5）		
危险度分层［例（%）］			*χ^2^*＝0.481	0.488
标危	5（11.9）	9（17.0）		
高危	37（88.1）	44（83.0）		
移植时疾病状态［例（%）］			*χ^2^*＝0.854	0.355
非CR	5（11.9）	10（18.9）		
CR	37（88.1）	43（81.1）		
移植时MRD［例（%）］			*χ^2^*＝0.835	0.361
阳性	13（31.0）	12（22.6）		
阴性	29（69.0）	41（77.4）		
供者类型［例（%）］			Fisher	0.164
单倍体	34（81.0）	35（66.0）		
同胞全相合	7（16.7）	17（32.1）		
无关	1（2.4）	1（1.9）		
HLA匹配程度［例（%）］			*χ^2^*＝2.622	0.105
全相合	8（19.0）	18（34.0）		
单倍体	34（81.0）	35（66.0）		
造血干细胞来源［例（%）］			*χ^2^*＝3.042	0.081
骨髓+外周血干细胞	25（59.5）	22（41.5）		
外周血干细胞	17（40.5）	31（58.5）		
诊断至移植时间［例（%）］			*χ^2^*＝0.842	0.359
≥8个月	23（54.8）	24（45.3）		
<8个月	19（45.2）	29（54.7）		
MNC输注量［×10^8^/kg，*M*（范围）］	11.17（5.78~17.04）	10.12（5.80~17.97）	*z*＝−1.701	0.089
CD34^+^细胞输注量［×10^6^/kg，*M*（范围）］	3.04（1.00~14.55）	3.21（0.56~9.22）	*z*＝−0.472	0.637

注 T-ALL：急性T淋巴细胞白血病；B-ALL：急性B淋巴细胞白血病；CR：完全缓解；MRD：微小残留病；MNC：单个核细胞

二、造血重建

TBI/Cy组和Bu/Cy组粒细胞植入的中位时间分别为14（10～25）d、14（10～24）d；血小板植入的中位时间分别为17（10～42）d、19（11～42）d。两组患者全部实现粒细胞植入，TBI/Cy组2例患者和Bu/Cy组1例患者分别因GVHD、重度感染于移植后100 d内死亡，未完成血小板植入。两组患者粒细胞及血小板植入情况比较，差异无统计学意义（均*P*>0.05）。详见表2。

**表2:** 两种异基因造血干细胞移植预处理方案急性淋巴细胞白血病患者的移植结局比较

移植特征	Bu/Cy组（42例）	TBI/Cy组（53例）	统计量	*P*值
粒细胞植入时间［d，*M*（范围）］	14（10~24）	14（10~25）	*z*＝−1.614	0.106
血小板植入时间［d，*M*（范围）］	19（11~42）	17（10~42）	*z*＝−0.693	0.488
Ⅱ~Ⅳ度aGVHD［例（%）］	15（35.7）	22（41.5）	*χ^2^*＝0.331	0.565
Ⅲ/Ⅳ度aGVHD［例（%）］	2（4.8）	13（24.5）	*χ^2^*＝6.885	0.009
重度cGVHD［例（%）］	5（13.5）	8（16.7）	*χ^2^*＝0.160	0.689
CMV血症［例（%）］	15（35.7）	22（41.5）	*χ^2^*＝0.331	0.565
EBV血症［例（%）］	15（35.7）	18（34.0）	*χ^2^*＝0.032	0.859
重度感染［例（%）］	14（33.3）	23（43.4）	*χ^2^*＝0.998	0.318
出血性膀胱炎［例（%）］	21（50.0）	11（20.8）	*χ^2^*＝8.972	0.003

注 aGVHD：急性移植物抗宿主病；cGVHD：慢性移植物抗宿主病；Bu：白消安；Cy：环磷酰胺；TBI：全身放射治疗；CMV：巨细胞病毒；EBV：EB病毒；

三、GVHD

TBI/Cy组、Bu/Cy组aGVHD发生率分别为52.8％、47.6％（*χ*^2^＝0.002，*P*＝0.965）。其中Ⅱ～Ⅳ度aGVHD发生率分别为41.5％、35.7％（*χ*^2^＝0.331，*P*＝0.565），Ⅲ/Ⅳ度aGVHD发生率分别为24.5％、4.8％（*χ*^2^＝6.885，*P*＝0.009）。10例患者于移植后100 d内死亡，对其余85例评估cGVHD，两组cGVHD发生率分别为50.0％、43.2％（*χ*^2^＝0.383，*P*＝0.536），其中重度cGVHD发生率分别为16.7％、13.5％（*χ*^2^＝0.160，*P*＝0.689）（表2）。

四、感染及其他并发症

TBI/Cy组、Bu/Cy组CMV血症的发生率分别为41.5％、35.7％（*χ*^2^＝0.331，*P*＝0.565），EBV血症的发生率分别为34.0％、35.7％（*χ*^2^＝0.032，*P*＝0.859）；感染的发生率分别为62.3％、47.6％（*χ*^2^＝2.038，*P*＝0.153），其中重度感染的发生率分别为43.4％、33.3％（*χ*^2^＝0.998，*P*＝0.318）。两组出血性膀胱炎发生率分别为20.8％、50.0％（*χ*^2^＝8.972，*P*＝0.003）。详见表2。

五、复发与生存

TBI/Cy、Bu/Cy组中位随访时间分别为37.1（2.7～97.3）、53.3（2.0～95.8）个月（*χ*^2^＝0.150，*P*＝0.698），移植后2年累积复发率分别为17.0％、42.9％（*χ*^2^＝5.654，*P*＝0.017）；2年NRM分别为24.5％、7.1％（*χ*^2^＝2.418，*P*＝0.120）；2年DFS率分别为58.5％、50.0％（*χ*^2^＝0.530，*P*＝0.466）；2年OS率分别为69.8％、64.3％（*χ*^2^＝0.152，*P*＝0.697）。生存曲线见图1。

**图1:**
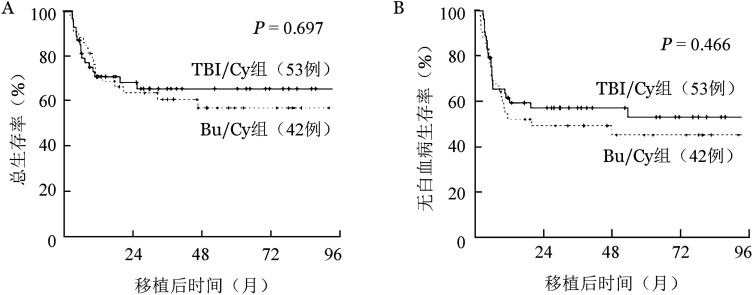
全身放射治疗/环磷酰胺（TBI/Cy）和白消安/环磷酰胺两种预处理方案组移植后总生存曲线（A）和无白血病生存曲线（B）

六、预后因素分析

单因素分析显示，影响复发的因素有预处理方案、诊断时外周血高白细胞、移植时疾病缓解状态及移植时MRD；影响NRM的因素有疾病分型、移植时MRD、发生Ⅲ/Ⅳ度aGVHD、髓外浸润史及感染；影响DFS的因素有诊断时外周血高白细胞、移植时疾病缓解状态、移植时MRD及HLA相合程度；影响OS的因素有诊断时外周血高白细胞、移植时MRD、发生Ⅲ/度aGVHD及感染。多因素分析显示，基于TBI/Cy的预处理方案是移植后复发的保护性因素（*HR*＝0.304，*P*＝0.004），诊断时外周血高白细胞（*HR*＝2.521，*P*＝0.021）、移植时疾病非CR（*HR*＝5.483，*P*＝0.002）是移植后复发的危险因素；发生Ⅲ/Ⅳ度aGVHD（*HR*＝4.388，*P*＝0.009）及感染（*HR*＝5.462，*P*＝0.047）是非复发死亡的危险因素；诊断时外周血高白细胞（*HR*＝2.412，*P*＝0.005）、移植时疾病非CR（*HR*＝2.749，*P*＝0.016）是影响DFS的危险因素；感染是影响OS的独立危险因素（*HR*＝2.480，*P*＝0.037）（表3）。

**表3:** 成人急性淋巴细胞白血病异基因造血干细胞移植后复发、非复发死亡及生存的单因素与多因素分析

影响因素	单因素分析		多因素分析	
	*HR*（*95%CI*）	*P*值	*HR*（*95%CI*）	*P*值
复发				
预处理方案（TBI/Cy，Bu/Cy）	0.407（0.189~0.876）	0.021	0.304（0.135~0.688）	0.004
诊断时高白细胞血症（是，否）	2.878（1.332~6.217）	0.007	2.521（1.151~5.521）	0.021
移植时疾病缓解状态（非CR，CR）	4.886（2.170~11.001）	0.001	5.483（1.909~15.746）	0.002
移植时MRD（阳性，阴性）	3.722（1.759~7.877）	0.001		
NRM				
预处理方案（TBI/Cy，Bu/Cy）	2.608（0.743~9.161）	0.135	1.393（0.355~5.462）	0.634
疾病免疫分型（T-ALL，B-ALL）	2.850（1.060~7.662）	0.038		
髓外浸润病史（有，无）	2.357（0.876~6.337）	0.089		
移植时MRD（阳性，阴性）	2.855（0.986~8.262）	0.053		
Ⅲ/Ⅳ度aGVHD（有，无）	4.978（1.863~13.306）	0.001	4.388（1.451~13.272）	0.009
感染（有，无）	6.073（1.378~26.761）	0.017	5.462（1.024~29.135）	0.047
LFS				
预处理方案（TBI/Cy，Bu/Cy）	0.805（0.449~1.445）	0.467	0.794（0.430~1.468）	0.462
诊断时高白细胞血症（是，否）	2.447（1.335~4.485）	0.004	2.412（1.297~4.487）	0.005
移植时疾病缓解状态（非CR，CR）	3.297（1.648~6.597）	0.001	2.749（1.209~6.251）	0.016
移植时MRD（阳性，阴性）	2.906（1.584~5.329）	0.001		
HLA匹配程度（全相合，单倍体）	0.498（0.232~1.070）	0.074		
OS				
预处理方案（TBI/Cy，Bu/Cy）	0.875（0.446~1.715）	0.697	0.698（0.335~1.456）	0.338
诊断时高白细胞血症（是，否）	2.982（1.446~6.147）	0.003		
移植时MRD（阳性，阴性）	2.090（1.030~4.242）	0.041		
Ⅲ/Ⅳ度aGVHD（有，无）	2.555（1.188~5.493）	0.016		
感染（有，无）	3.135（1.414~6.950）	0.005	2.480（1.055~5.828）	0.037

注 TBI：全身放射治疗；Cy：环磷酰胺；Bu：白消安；CR：完全缓解；NRM：非复发死亡；LFS：无白血病生存；OS：总生存；MRD：微小残留病；aGVHD：急性移植物抗宿主病；cGVHD：慢性移植物抗宿主病

## 讨论

ALL是成人最常见的急性白血病之一，恶性程度高，预后差[1]。尽管ALL患者通常对于化疗敏感，缓解率很高，但超过半数患者会出现复发，复发的患者往往远期预后差[16]–[18]。allo-HSCT可以显著降低复发率，提高患者长期生存率，仍是成人ALL患者的唯一治愈性手段[2],[18]–[20]。预处理方案是allo-HSCT的重要组成部分，已被证明会影响HSCT的疗效及白血病患者的预后。基于TBI和基于白消安的清髓性预处理方案，是ALL患者allo-HSCT最常用的两种标准预处理方案[18],[21]–[22]。两种方案对成人ALL患者allo-HSCT疗效及预后的影响，仍存在很大争议。

ALL患者移植失败的最主要原因是复发[23]–[24]，影响移植后复发的因素多种多样，而预处理方案在预防复发中起到了重要作用[25]。既往一些研究表明，基于口服白消安的预处理方案比基于TBI的方案，移植后复发率更高[5],[26]。随着白消安静脉制剂的应用，基于TBI的预处理方案降低移植后复发风险的优势存在争议。有一些研究认为，白消安静脉注射给药具有稳定的药物代谢动力学，且可透过血脑屏障，从而具有与TBI方案相似的复发率[8],[27]。也有一些研究认为，基于TBI的预处理方案比基于白消安静脉制剂的方案复发率显著降低[4]–[5],[7],[26]。然而以上结果均来自于回顾性研究，在Zhang等[10]对于550例CR1期行allo-HSCT的成人标危B-ALL患者的RCT研究中，Bu/Cy和TBI/Cy预处理方案组移植后2年复发率相似（20.2％对18.4％）。而近年来Peters等[6]的另一项多中心随机对照研究中，纳入了417例移植前完全缓解的高危儿童ALL患者，比较了TBI联合依托泊苷与化疗预处理，发现TBI预处理和化疗预处理后两年累积复发率分别为12％、33％（*P*<0.001），认为TBI方案移植后复发风险更低，这与我们的结论一致。在我们的研究中，成人ALL患者接受allo-HSCT治疗时，应用含TBI的预处理方案比基于白消安静脉给药方案移植后复发率明显降低（*HR*＝0.304，95％*CI* 0.135～0.688，*P*＝0.004）。认为可能与TBI发挥更强的抗肿瘤活性，从而降低了移植后复发的风险有关。另外，在我们的研究中，移植后复发率与Peters等[6]的研究相近，但高于Zhang等[10]的研究，认为有可能与我们纳入的患者为中高危、复发/难治者较多有关。

耐受性是选择预处理方案的另一个重要因素。预处理方案强度的提高，在使疾病复发率明显降低的同时，往往造成组织损伤，增加非复发死亡风险。既往一些研究认为，与基于白消安的预处理相比，基于TBI的方案降低移植后复发率的优势往往被NRM所抵消[7],[28]–[29]。EBMT的一项研究表明，基于TBI的预处理方案不能为年龄≥35岁的T-ALL患者带来任何生存获益，因为与基于白消安的方案相比，NRM的风险更高（38％对9％，*P*＝0.01）[30]。而Shigematsu等[31]研究显示基于TBI（12 Gy）的预处理方案治疗成人ALL患者，在不增加NRM的情况下，实现了疾病的良好控制。Zhang等[10]的研究中，TBI预处理组与化疗预处理组两年NRM相似。而Peters等[6]的研究中，化疗预处理组两年NRM显著高于TBI组。在我们的研究中，多因素分析显示，基于TBI的预处理方案，NRM风险高于Bu/Cy方案（*HR*＝1.393，95％*CI* 0.355～5.462），但不具有统计学意义（*P*＝0.634）。因此我们认为，含TBI的预处理方案对于降低移植后复发率而不增加NRM发挥了重要作用。

我们的研究还发现，与仅基于化疗的预处理方案相比，含TBI的方案Ⅲ/Ⅳ度aGVHD的发生率明显增加（24.5％对4.8％，*P*＝0.009）。这与既往一些研究相一致[4],[32]–[33]。近年来，随着移植后环磷酰胺（PTCy）的广泛应用，被证实可以降低GVHD的发生风险[34]–[36]，Dholaria等[33]研究发现和PTCy联合使用时，基于TBI的预处理方案与Ⅲ/Ⅳ度aGVHD的发生风险无关，NRM较低，认为可能是由于在联合PTCy预防GVHD的情况下，基于TBI的预处理方案可导致更好的免疫重建。随着支持性治疗的进步，应用新型GVHD预防方案与基于TBI的预处理方案相结合，或许有助于改善TBI方案的GVHD结局。

一些研究认为基于TBI预处理方案的患者，移植后总生存率要优于基于白消安的方案[5],[30]，也有一些研究表明两种方案移植后OS率相当[7]–[8],[27]。两项RCT研究中，Zhang等[10]发现对于CR1期成人标危B-ALL患者，Bu/Cy方案和TBI/Cy方案观察到相似的OS率和DFS率；而Peters等[6]对于CR期高危儿童ALL患者的研究中，TBI联合依托泊苷预处理后生存率比仅化疗方案明显更高。在本研究中，含TBI预处理方案的患者，移植后2年OS、DFS与仅基于化疗方案的患者相当（OS：69.8％对64.3％，*χ*^2^＝0.152，*P*＝0.697；DFS：58.5％对50.0％，*χ*^2^＝0.530，*P*＝0.466）。多因素分析中，含TBI的预处理方案也未能改善OS（*HR*＝0.698，95％*CI* 0.335～1.456，*P*＝0.338）及DFS（*HR*＝0.794，95％*CI* 0.430～1.468，*P*＝0.467）。在多因素分析中，感染是影响ALL患者移植后OS的独立危险因素（*HR*＝2.480，95％*CI* 1.055～5.828，*P*＝0.037）。而含TBI的预处理方案，加强免疫抑制的同时，感染的风险提高，从而可能影响OS。本研究中，基于TBI的方案，移植后复发率显著降低，但未能转化为OS的改善，我们认为可能和感染及重度aGVHD的发生风险提高有关。此外，也可能受病例数量的限制，以及回顾性研究偏倚的影响。随着支持性治疗的进步，在应用含TBI方案的患者中加强感染的预防和控制并联合应用新型的GVHD预防方案，也许可以在控制疾病、降低复发率的同时，进一步降低非复发死亡风险，从而转化为更好的OS及DFS，充分发挥TBI抗白血病作用的优势。
